# Arbuscular Mycorrhizal Fungi Mediate the Acclimation of Rice to Submergence

**DOI:** 10.3390/plants13141908

**Published:** 2024-07-10

**Authors:** Yanggui Xu, Yuting Tu, Jiayi Feng, Zhiping Peng, Yiping Peng, Jichuan Huang

**Affiliations:** 1 Institute of Agricultural Resources and Environment, Guangdong Academy of Agricultural Sciences, Guangzhou 510640, China; xuyanggui@gdaas.cn (Y.X.); tuyuting@gdaas.cn (Y.T.); pengzhiping@gdaas.cn (Z.P.); pengyiping@gdaas.cn (Y.P.); 2 Key Laboratory of Plant Nutrition and Fertilizer in South Region, Ministry of Agriculture, Guangzhou 510640, China; 3 Guangdong Key Laboratory of Nutrient Cycling and Farmland Conservation, Jinying Road, Guangzhou 510640, China; 4 Guangdong Eco-Engineering Polytechnic, Guangzhou 510520, China; leave4s@126.com

**Keywords:** carbohydrates, lipids, fungal perception, root morphological traits, flooding stress, submergence-related genes

## Abstract

Flooding is a critical factor that limits the establishment of a symbiosis between rice and arbuscular mycorrhizal fungi (AMF) in wetland ecosystems. The distribution of carbon resources in roots and the acclimation strategies of rice to flooding stress in the presence of AMF are poorly understood. We conducted a root box experiment, employing nylon sheets or nylon meshes to create separate fungal chambers that either prevented or allowed the roots and any molecules to pass through. We found that the mycorrhizal colonization rate and the expression of genes *OsD14L* and *OsCERK1*, which are involved in fungal perception during symbiosis, both increased in mycorrhizal rice roots following intermittent flooding compared to continuous flooding. Furthermore, AMF inoculation affected root morphological traits, facilitating both shallower and deeper soil exploration. Increased submergence intensity led to carbohydrate deprivation in roots, while high mycorrhizal colonization increased soil oxygen consumption and decreased the neutral lipid concentration in roots. However, mycorrhizal inoculation increased the rice photosynthesis rate and facilitated acclimation to submergence by mediating the expression of the genes *OsCIPK15* and *OsSUB1A* to enhance rice shoot elongation and the sugar concentration in roots as a result of reduced competition for carbon between rice and AMF under different flooding conditions.

## 1. Introduction

Arbuscular mycorrhizal fungi (AMF) form mutualistic associations with more than 80% of all vascular plant species, including many important food crops [1]. These symbiotic interactions provide nutrients for plants, thereby increasing crop productivity as well as plant tolerance [2,3,4]. Rice (*Oryza sativa*) is one of the most important food crops globally and is traditionally cultivated under flooding conditions [5,6]. It has been used as one of the model plants for understanding the biochemical and physiological basis of AMF symbiosis in monocotyledonous plants [7,8].

The successful colonization of rice by AMF is related to several key factors. It has been reported that *OsMYR1* and *OsCERK1* encode key receptors for signals from AMF during symbiosis; OsMYR1 can directly bind to the signaling molecule CO_4_, secreted by AMF, and this binding promotes the dimerization and phosphorylation of the OsMYR1/OsCERK1 receptor complex, thereby activating downstream symbiotic signaling pathways [9]. The a/b-fold hydrolase DWARF14LIKE (D14L) also participates in pre-symbiotic fungal perception, which is essential for symbiosis [10]. It has traditionally been thought that most wetland plants under flooding conditions are non-mycorrhizal or have low colonization rates [11,12,13,14]. Although AMF are common fungi in paddy soil, the existence of AMF and their possible practical role in wetland systems have been ignored [15,16,17,18].

In the last two decades, increasing evidence has accumulated showing that AMF can form a symbiosis with many wetland plants under different hydrological conditions [7,13,19]. Symbiotic interactions between plants and AMF rely on a complex molecular dialog with reciprocal benefits in terms of nutrition, growth, and protection [20]. Membrane lipids, key substances for symbiosis, cannot be synthesized by AMF themselves and must be obtained from host plants [10,21,22]. Plants transfer 10–30% of their photosynthetically fixed carbon, in the form of sugars and lipids, to AMF in exchange for nutrients from AMF, especially phosphorus [23]. However, traditional rice cultivation under flooding consumes 80% of agricultural freshwater resources [24]. In particular, continuous flooding can lead to greater carbohydrate consumption, peroxidation of cell membranes, and low photosynthetic rates of plants due to limited availability of oxygen [25,26]. It has been reported that flooding intensity can effectively affect the outcome of AMF symbiosis for rice plants [27]. Intermittent flooding, a common water-saving technique in irrigated rice cultivation, has been shown to maintain grain yield as effectively as continuous flooding [28]. However, the distribution of carbon resources in roots and the acclimation of rice to different flooding conditions in the presence of AMF are poorly understood.

The gene *CIPK15*, involved in a ‘submergence metabolic-acclimation strategy’, regulates carbon metabolism and rice growth under flooding, while the gene *SUB1A* is involved in a ‘submergence-escape strategy’, mediating gibberellic acid (GA) action to promote shoot elongation to allow rice shoots to rapidly rise above the floodwater surface [29]. Considering the obligate symbiotic nature of AMF and their requirement for oxygen to survive, we hypothesized that rice plants might mediate the AMF symbiosis extent and activate a submergence-acclimation strategy, thereby reducing carbon competition with AMF and improving their acclimation to stress at different intensities of submergence. Elucidating the mechanism of the symbiosis between AMF and rice is crucial. It helps in understanding how photosynthetically fixed carbon is allocated in mycorrhizal rice roots under both continuous and intermittent flooding conditions. This knowledge can contribute to water conservation, regulate rice acclimation to varying degrees of flooding stress, and promote the sustainable development of rice production.

## 2. Materials and Methods

### 2.1. Study Site

The experiment was conducted in a greenhouse at the Institute of Agricultural Resources and Environment, Guangdong Academy of Agricultural Sciences, Guangzhou, China from March to July 2023. The region has a subtropical monsoon climate with mean minimum and maximum temperatures in summer of 28 and 34 °C, respectively. The rice variety, Meixiangzhan 2 (*Oryza sativa*), is supplied by the Rice Research Institute of Guangdong, Guangzhou, China. An AMF *Rhizophagus irregularis* inoculant, which was provided by the College of Forestry, Northwest A&F University, Yangling, China, is widely distributed in paddy fields [30].

### 2.2. Root Box Experiment 

**Figure 1:**
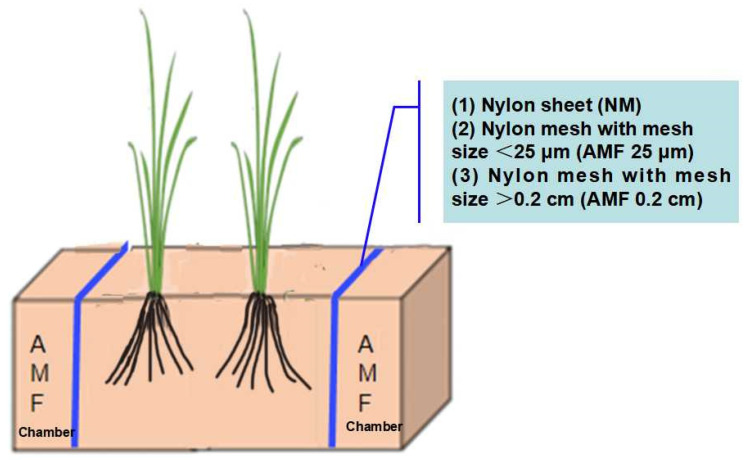
Arbuscular mycorrhizal fungi (AMF) chamber—Root box diagram. Treatments: controls received no inoculum in the AMF chamber, with a nylon sheet preventing plant roots and mycorrhizal hyphae from passing through and preventing diffusion of substances (NM); AMF chamber with nylon mesh with a diameter <25 μm preventing plant roots and mycorrhizal hyphae from passing through, but allowing diffusion of substances (AMF 25 μm); AMF chamber with nylon mesh with a diameter >0.2 cm, allowing plant roots and mycorrhizal hyphae to pass through and also allowing substances to diffuse (AMF 0.2 cm). Flooding management comprised continuous flooding (Con) and intermittent flooding (Int).

The root box experiment was conducted in plastic pots (30 cm in length, 15 cm in width, and 20 cm in height) containing a 3.5 kg 3:1 (*w*/*w*) mixture of autoclaved paddy soil and sand. The soil properties were: pH 6.59, organic matter content 22.5 g kg^−1^, available nitrogen (N) 136.9 mg kg^−1^, available phosphorus (P) 12.1 mg kg^−1^, and available potassium (K) 88.6 mg kg^−1^. Each root box employed nylon sheets or nylon meshes to create two separate fungal chambers on both sides that either prevented or allowed the roots and any molecules to pass through. The area between the two fungal chambers (the central area of the root box) served as the rice cultivation area (Figure 1). Each fungus chamber added 50 g of the fungal inoculum. Non-mycorrhizal (NM) controls received no inoculum, using nylon sheets to prevent the passage of plant roots and diffusing substances through the fungus chambers. The AMF 25 μm treatments employed nylon meshes with a mesh size < 25 μm, precluding the passage of roots and mycorrhizal hyphae but allowing diffusion of substances through the fungus chambers. Conversely, the AMF 0.2 cm treatments used nylon meshes with a mesh size > 0.2 cm, allowing root passage and substance diffusion. Rice seeds were sterilized with a 10 % (*v*/*v*) solution of hydrogen peroxide and were subsequently germinated in the autoclaved sand. All seedlings were cultivated in a greenhouse. Two seedlings were transferred to the rice cultivation area in each root box and planted 10 cm apart. Flooding management comprised continuous flooding (water level above the topsoil was 2–4 cm; Con) and intermittent flooding (alternate drainage and flooding every seven days, and the soil was kept moist after drainage; Int) and each treatment had four replicates. Plant harvesting was at the late tillering stage, where one plant was used for analysis of root morphology and AMF colonization, and the other was used for determining root lipids, carbohydrates, and genes related to O_2_ deficiency and fungal perception. Plant height was measured by a ruler, and photosynthesis was measured by a portable infrared analyzer (Cl-340, CID, Inc., Camas, WA, USA).

### 2.3. Root Morphological Traits

In the root box experiment, the whole roots of one plant of each treatment were separated from shoots and placed in individual plastic bags after washing the roots from soil particles. These roots were kept at 4 °C for further processing. The root systems were scanned using a Scanner Expression 120000XL (Regent Instruments Inc., Quebec, QC, Canada) to obtain a 2400 dpi image. The lateral roots (LRs)’ surface area, volume, length, mean diameter, and number of branching roots (BRs) were determined using the WinRHIZO LA 2400 system (Regent Instruments Inc., Quebec, QC, Canada). S-type (short, thin, and capable of absorption) and L-type (long, thick, and capable of further branching) were separated by the LRs’ length per diameter class [31].

### 2.4. Estimation of Root Colonization by AMF

Root samples were cleared in 10% (*w*/*v*) KOH at 100 °C for 3–5 min, and then rinsed several times with tap water following staining with 5% ink-vinegar solution (*v*/*v*) for 3 min [32]. Roots were destained by rinsing with deionized water (acidified with a few drops of vinegar) for a minimum of 20 min and kept in a tube with 1% vinegar solution until root colonization by AMF was quantified by the grid crossing method [33].

### 2.5. Lipid and Carbohydrate Component Analysis

The lipids were extracted from LRs, basically following the method described by Frostegård et al. [34]. Briefly, freeze-dried, crushed LR samples (0.25 g) were placed in a tube containing a one-phase mixture of citrate buffer, chloroform, and ethanol (0.8:1:2 *vol*/*vol*/*vol*; pH 4.0) at room temperature for at least 2 h. The tubes were centrifuged at 400× *g* for 15 min, and the supernatants were then transferred to new test tubes. The residues were washed with 5 mL of the single-phase mixture. The neutral lipids and phospholipids in combined supernatants were determined using the Lipid Quantification Kit (Cell Biolabs, Inc., San Diego, CA, USA) and Plant Phospholipid ELISA Kit (Jining Shiye, Shanghai, China) according to the manufacturer’s instructions after the organic solvent was removed under a stream of nitrogen. Total soluble sugar and starch concentrations were measured by the phenol-sulfuric acid and anthrone methods, respectively [35,36].

### 2.6. Quantitative Real-Time PCR (qRT-PCR) Analysis

Total RNA was extracted from frozen-flesh LRs using the TRUEscript RT MasterMix (for real-time PCR) (DF Biotech, Chengdu, China). First-strand cDNA was synthesized from 0.5 µg of total RNA using the TransScript First-Strand cDNA Synthesis kit (AiDLAB Biotech, Beijing, China) according to the manufacturer’s instructions. UBI was employed as an internal reference gene, and quantitative real-time PCR (qRT-PCR) was then performed on a CFX96 Real-Time PCR Detection System (Bio-Rad, Hercules, CA, USA) using the SYBR Green QPCR Mix (DF Biotech, Chengdu, China). The relative expression levels of genes were calculated using the 2^−△△Ct^ method [37]. Sequences of gene-specific primers are listed in Supplementary Table S1. The sequence data were deposited in the database of the Rice Genome Annotation Project under the following accession: *OsCERK1* (LOC_Os08g42580), *OsD14L* (LOC_Os03g32270), *OsMYR* (LOC_Os03g13080), *OsSUB1A* (DQ011598), *OsCIPK15* (LOC_Os11g02240), *OsUBI* (LOC_Os03g13170).

### 2.7. Soil Chemical Properties

Soil dissolved oxygen (DO) was measured using the Thermo RDO Portable Meter (Scientifific™ Orion™ Star A329, Woonsocket, RI, USA) through a plastic tube (diameter, 1.5 cm; height, 25 cm) inserted into the soil near the rhizosphere of each plant, as described previously [38]. Soil pH was measured using a pH meter at a soil-to-deionized water ratio of 1:2.5 (*w*/*v*). Available N was measured by the alkali solution diffusion method, and available K was measured by flame photometry after extraction with 1 MNH_4_OAc [39]. Available P was measured by a spectrophotometer using the colorimetric method, and organic carbon concentration was measured by the potassium dichromate oxidation heating method [39,40].

### 2.8. Statistical Analyses

Data were analyzed using SPSS statistics 21.0 for *t*-tests, one-way ANOVA, and two-way ANOVA. The statistical significance level of the differences tested among the experimental data is *p* < 0.05 unless stated otherwise. Figures were drawn by SigmaPlot 14.0 and Visio 2013.

## 3. Results

### 3.1. Dissolved Oxygen in Soil

In non-mycorrhizal (NM) controls, soil DO increased following intermittent flooding compared with continuous flooding. Under intermittent flooding, soil DO decreased following the AMF 0.2 cm treatment compared to the NM treatment, but there were no differences between continuous flooding and intermittent flooding in AMF 0.2 cm treatments (Table S2).

### 3.2. Variations in Colonization Rate of AMF and Expression of Rice Genes under Different Treatments

The mycorrhizal colonization rate in roots increased following the reduction in the flooding intensity (Table S2). Rice roots under intermittent flooding (27.9%) showed about 3.7 times more mycorrhizal colonization than under continuous flooding (8.0%).

**Figure 2:**
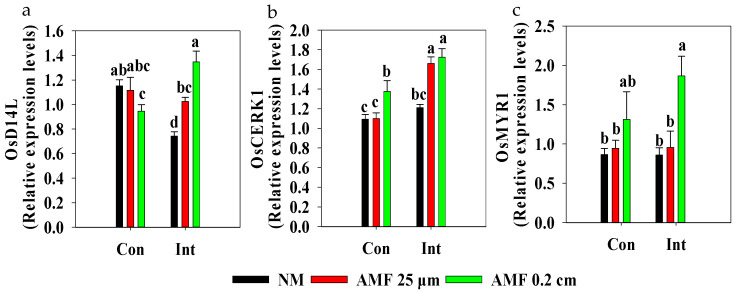
Effect of arbuscular mycorrhizal fungi (AMF) on the expression of rice genes involved in fungal perception under different flooding conditions. Abbreviations as in Figure 1. Different lowercase letters indicate significant differences among treatments at *p* < 0.05. (**a**) *OsD14L* in roots; two-way ANOVA, *F*_AMF_ = 4.452, *p* = 0.034; *F*_interaction_ = 18.754, *p* < 0.001. (**b**) *OsCERK1* in roots; two-way ANOVA, *F*_flooding_ = 23.693, *p* < 0.001; *F*_AMF_ = 10.709, *p* = 0.002; *F*_interaction_ = 3.278, *p* = 0.07. (**c**) *OsMYR1* in roots; two-way ANOVA, *F*_AMF_ = 6.015, *p* = 0.016.

We also investigate the effect of different intensities of flooding on rice genes *OsCERK1*, *OsMYR*, and *OsD14L*, which are involved in perceiving signals from AMF during symbiosis (Figure 2). The expression of *OsD14L* in roots was down-regulated following intermittent flooding compared with that under continuous flooding in the NM treatments and was down-regulated following AMF 0.2 cm mesh treatment compared with that of NM treatment under continuous flooding (Figure 2a). Conversely, AMF 25 μm mesh and AMF 0.2 cm mesh treatments generally up-regulated the expression of *OsD14L* in roots under intermittent flooding. There were no differences in the expression of *OsCERK1* and *OsMYR* in roots between continuous flooding and intermittent flooding in NM treatments, but an increase of *OsCERK1* expression was observed in AMF 0.2 cm mesh treatments compared with that of NM treatments under continuous flooding and intermittent flooding (Figure 2b,c). Particularly, the AMF 0.2 cm mesh treatment had a higher *OsCERK1* expression in roots under intermittent than under continuous flooding. It also showed an increase of *OsMYR* expression in roots of AMF 0.2 cm mesh treatment and an increase of *OsCERK1* expression in roots of AMF 25 μm mesh treatment compared to NM treatment under intermittent flooding.

**Figure 3:**
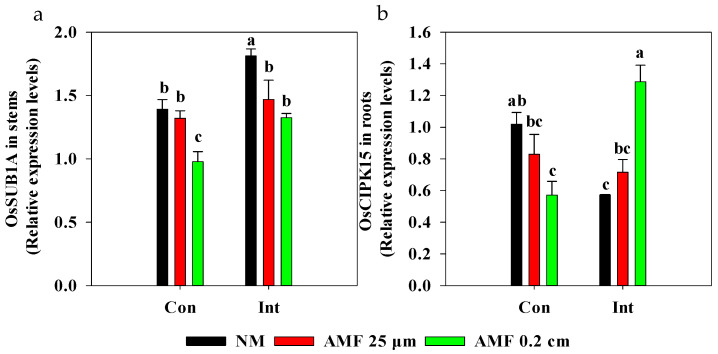
Effects of arbuscular mycorrhizal fungi (AMF) on the expression of genes response to submergence in rice roots. Abbreviations as in Figure 1. Different lowercase letters indicate significant differences among treatments at *p* < 0.05. (**a**) *OsSUB1A* in stems; two-way ANOVA, *F*_flooding_ = 15.690, *p* = 0.002; *F*_AMF_ = 11.390, *p* = 0.002; *F*_interaction_ = 1.123, *p* = 0.357. (**b**) *OsCIPK15* in roots; two-way ANOVA, *F*_interaction_ = 19.342, *p* < 0.001.

Intermittent flooding reduced flooding intensity and alleviated the O_2_ deficiency near rice roots (Table S2). A major submergence-tolerance QTL, with *OsSub1A* in the stem, was up-regulated following intermittent flooding in NM treatments (Figure 3a). However, the expression of *OsSub1A* was down-regulated following the AMF 0.2 cm mesh treatments under intermittent flooding and continuous flooding conditions. We also found a decrease in *OsSub1A* expression in the AMF 25 μm mesh treatment compared to that in the NM treatment under intermittent flooding. *OsCIPK15* in roots was down-regulated following intermittent flooding in NM treatments (Figure 3b). The AMF 0.2 cm mesh treatment exhibited a down-regulated expression of *OsCIPK15* in roots compared to that in the NM treatment under continuous flooding. In contrast, in the AMF 0.2 cm mesh treatment, the expression of *OsCIPK15* in roots was up-regulated compared to that in the NM treatment under intermittent flooding. The AMF 0.2 cm mesh treatment led to a higher *OsCIPK15* expression in the roots under intermittent flooding than under continuous flooding.

### 3.3. Variations in Rice Photosynthesis, Plant Height, and Root Morphological Traits under Different Treatments

**Figure 4:**
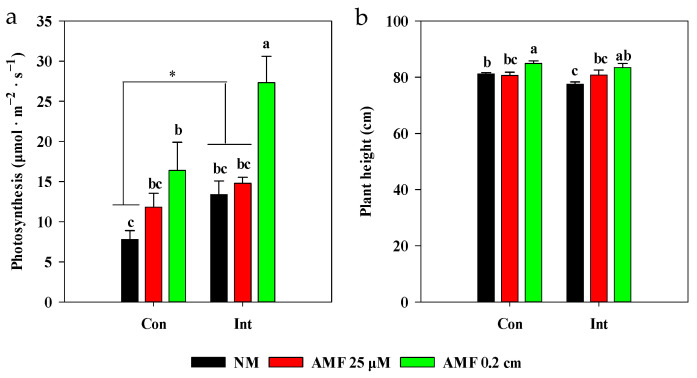
Effects of arbuscular mycorrhizal fungi (AMF) on photosynthesis and height of rice under different flooding conditions. Abbreviations as in Figure 1. Different lowercase letters indicate significant differences among treatments at *p* < 0.05. * Indicates significant differences at *p* < 0.05, *t*-test. (**a**) Photosynthesis of rice; two-way ANOVA, *F*_flooding_ = 12.375, *p* = 0.004; *F*_AMF_ = 13.508, *p* = 0.001; *F*_interaction_ = 1.606, *p* = 0.241. (**b**) Height of rice; two-way ANOVA, *F*_flooding_ = 2.963, *p* = 0.1118; *F*_AMF_ = 8.655, *p* = 0.005; *F*_interaction_ = 1.269, *p* = 0.316.

In the root box experiment, intermittent flooding increased photosynthesis, but decreased plant height compared with continuous flooding in the NM treatments (Figure 4). However, both continuous flooding and intermittent flooding increased plant photosynthesis and plant height following AMF 0.2 cm mesh treatments compared with NM treatments.

**Figure 5:**
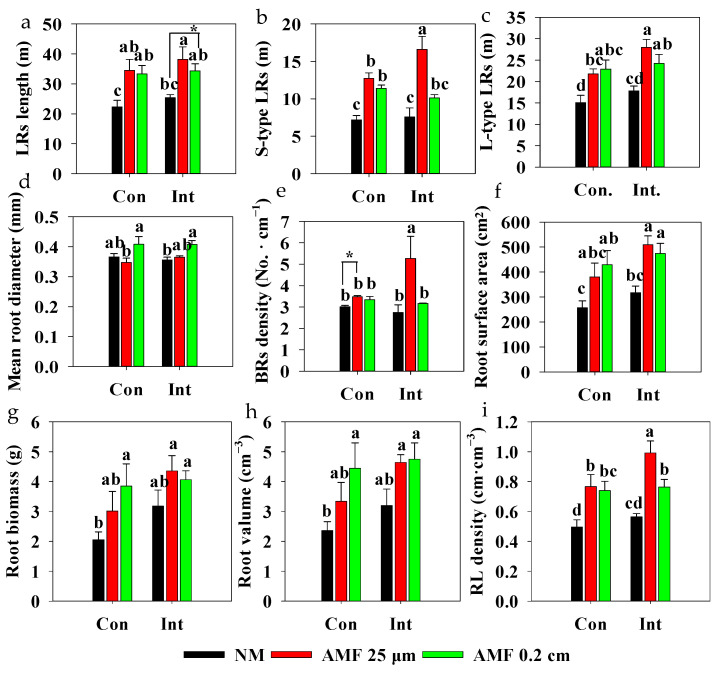
Effect of arbuscular mycorrhizal fungi (AMF) on root morphological traits of rice under different flooding conditions. Abbreviations as in Figure 1. Different lowercase letters indicate significant differences among treatments at *p* < 0.05. * Indicates significant differences at *p* < 0.05, *t*-test. (**a**) Length of the lateral roots (LRs); (**b**) Length of S-type LRs; (**c**) Length of L-type LRs; (**d**) Mean diameter of roots; (**e**) Branch density of roots; branching roots (BRs); (**f**) Surface area of roots; (**g**) Biomass of roots; (**h**) Valume of roots; (**i**) Density of root length (RL).

There was no significant difference in the lateral root (LR) morphology between continuous flooding and intermittent flooding in NM treatments (Figure 5). LR length, S-type LR length, L-type LR length, BR density, and root length (RL) density were increased in the AMF 25 μm treatments under continuous flooding and intermittent flooding compared with those of NM treatments, and the root surface area increased following AMF 25 μm mesh treatment under intermittent flooding (Figure 5a–d,f and Figure S1). AMF 25 μm mesh showed higher values of S-type LR length and RL density of rice under intermittent flooding than those of rice under continuous flooding. AMF 0.2 cm mesh treatments increased LR length, L-type LR length, root surface area, and RL density compared to those of NM treatments under continuous flooding and intermittent flooding. Additionally, AMF 0.2 cm mesh treatments increased S-type LR length under continuous flooding and increased mean root diameter under intermittent flooding compared to those in NM treatments (Figure 5). There were no differences in the root biomass and root volume between continuous flooding and intermittent flooding in the AMF 0.2 cm mesh treatments. However, under continuous flooding, we observed lower values of root biomass and volume in the NM treatment than in the AMF 0.2 cm mesh treatment.

### 3.4. Variations in Lipid Concentration in Rice Root Membrane under Different Treatments

**Figure 6:**
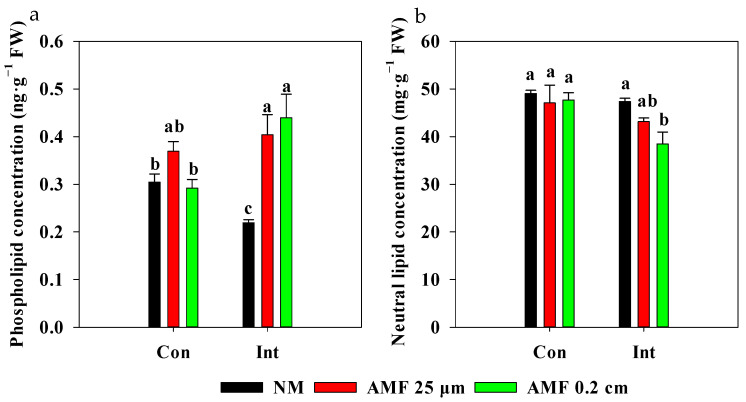
Effect of arbuscular mycorrhizal fungi (AMF) on the concentration of membrane lipids in roots under different flooding conditions. Abbreviations as in Figure 1. Different lowercase letters indicate significant differences among treatments at *p* < 0.05. (**a**) Concentration of phospholipid in rice roots; (**b**) Concentration of neutral lipid in rice roots.

Intermittent flooding decreased the phospholipid concentration in non-mycorrhizal roots compared to that of non-mycorrhizal roots under continuous flooding (Figure 6a). In contrast, the phospholipid concentration increased in roots following the AMF 0.2 cm mesh treatment and AMF 25 μm mesh treatment under intermittent flooding, and there was a higher phospholipid concentration in roots in the AMF 0.2 cm mesh treatment under intermittent flooding than that of the AMF 0.2 cm mesh treatment under continuous flooding. There was no difference in neutral lipid concentration in roots between the NM and AMF treatments under continuous flooding (Figure 6b). However, the AMF 0.2 cm mesh treatment decreased neutral lipid concentration in roots compared to that in the NM treatment under intermittent flooding and led to a lower neutral lipid concentration in the membrane of roots in the AMF 0.2 cm mesh treatment under intermittent flooding than that in the AMF 0.2 cm mesh treatment under continuous flooding.

### 3.5. Variations in Carbohydrate Concentrations in Rice Roots

**Figure 7:**
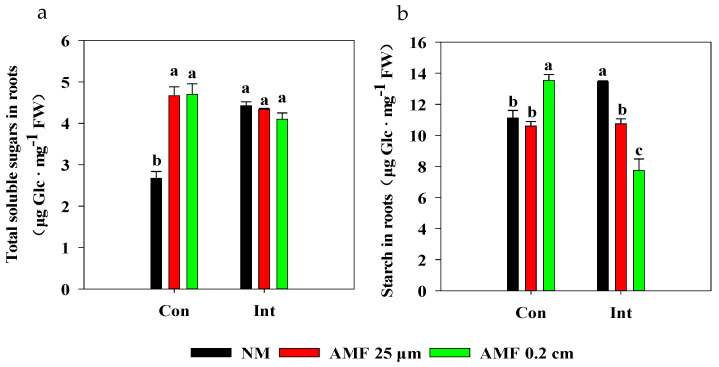
Effects of arbuscular mycorrhizal fungi (AMF) on carbohydrate concentrations in rice roots under different flooding conditions. Abbreviations as in Figure 1. Different lowercase letters indicate significant differences among treatments at *p* < 0.05. (**a**) Total soluble sugars in roots; two-way ANOVA, *F*_flooding_ = 3.98, *p* = 0.069; *F*_AMF_ = 19.011, *p* < 0.001, *F*_interaction_ = 28.473, *p* < 0.001. (**b**) Starch in roots; two-way ANOVA, *F*_flooding_ = 9.939, *p* = 0.008, *F*_AMF_ = 9.702, *p* = 0.003, *F*_interaction_ = 48.191, *p* < 0.001.

Intermittent flooding increased the total soluble sugar and starch concentrations in non-mycorrhizal roots compared to those in the non-mycorrhizal roots under continuous flooding (Figure 7). Total soluble sugar concentration in roots increased following the AMF 0.2 cm mesh and AMF 25 μm mesh treatments under continuous flooding, but there were no differences between the NM and AMF treatments under intermittent flooding (Figure 7a). However, in the AMF 0.2 cm mesh and AMF 25 μm mesh treatments, the starch concentration in roots decreased compared to that in non-mycorrhizal roots under intermittent flooding, especially in the AMF 0.2 cm mesh treatment with the lowest starch concentration (Figure 7b). In contrast, in the AMF 0.2 cm mesh treatment, the starch concentration in roots increased compared to that in non-mycorrhizal roots under continuous flooding.

## 4. Discussion

### 4.1. Effect of Arbuscular Mycorrhizal Fungi on Dissolved Oxygen in Soil

Traditional rice cultivation is conducted in wetland ecosystems, but continuous flooding inhibits mycorrhizal inoculation and rice growth [8]. In this study, intermittent flooding not only increased soil dissolved oxygen but also increased the mycorrhizal colonization rate in rice roots compared to that under continuous flooding (Table S2). These findings indicate that intermittent flooding decreases the submergence intensity and supports more oxygen to thrive the mycorrhizal inoculation in rice roots. However soil dissolved oxygen decreases following AMF 0.2 cm mesh treatment, with a higher mycorrhizal colonization rate under intermittent flooding. These observations suggest that increasing the mycorrhizal colonization rate in rice roots increases the consumption of oxygen.

### 4.2. Influence of Submergence on Fungal Perception, Arbuscular Mycorrhizal Fungi Colonization, and Concentration of Lipids in Root Membrane

*OsCERK1* is required to encode a co-receptor to activate AMF symbiosis signaling [41]. The AMF 0.2 cm mesh treatment led to a higher *OsCERK1* expression in roots under intermittent flooding than under continuous flooding (Figure 2b), suggesting that intermittent flooding might enhance the AMF symbiosis in roots (Table S2). Moreover, the gene product of *OsMYR1* can directly bind to the signaling molecule (the cocktail of chitinaceous compounds) secreted by AMF, and this binding promotes the dimerization and phosphorylation of the OsMYR1/OsCERK1 receptor complex, thereby activating downstream symbiotic signaling pathways [9,42]. There was no difference in *OsMYR1* expression between NM and AMF 0.2 cm mesh treatments under continuous flooding, but we observed an increase of *OsMYR1* expression in AMF 0.2 cm mesh treatment compared with the NM treatment under intermittent flooding (Figure 2c). These findings suggest that intermittent flooding might increase the secretion of signaling molecules (the cocktail of chitinaceous compounds) by AMF and activate downstream symbiotic signaling pathways, thereby promoting symbiotic relationships. Transcription of *OsD14L*, a gene encoding an intracellular α/β-fold hydrolase in rice roots responsible for the perception of AMF, was up-regulated following AMF 25 μm mesh treatment (preventing roots and mycorrhizal hyphae from passing through but allowing communication between substances) under intermittent flooding (Figure 2a). These findings suggest that exogenous AMF might trigger signaling in rice plants for pre-symbiotic fungal perception under intermittent flooding before establishing physical contact with rice [10]. *OsD14L* expression in roots in the AMF 0.2 cm mesh treatment (which allows the root to pass through fungus chambers) was up-regulated under intermittent flooding but down-regulated under continuous flooding compared to that in the NM or AMF 25 μm mesh treatments, demonstrating that physical contact could further trigger rice plant signaling for pre-symbiotic fungal perception under intermittent flooding, but that this was inhibited under continuous flooding. These findings suggest that physical contact might alter the oxygen or carbon requirement of AMF in the rice rhizosphere (Table S2 and Figure 7), which then affects the response of rice to fungal perception and the extent of colonization (Figure 2 and Table S2). This response might facilitate the AMF-rice system’s acclimation to varying degrees of oxygen deficiency under different flooding intensities.

Phospholipids are important constituents of biological membranes that act as signaling molecules, while neutral lipids are membrane components and are responsible for the storage of energy [43]. In the NM treatments, intermittent flooding decreased the phospholipid concentration in roots compared to that under continuous flooding (Figure 6a). This decrease might be attributed to intermittent flooding, reducing the *p* uptake by rice and leading to slower phospholipid synthesis in roots [44]. In contrast, the phospholipid concentration was increased in roots following AMF 25 μm mesh treatments under intermittent flooding. The AMF 25 μm mesh treatments prevented roots from passing through but allowed communication via chemical substances, further demonstrating that exogenous AMF might trigger endomembrane trafficking and signaling of pre-symbiotic fungal perception under intermittent flooding [45]. Rice in the AMF 0.2 cm mesh treatment had a higher phospholipid concentration in roots under intermittent flooding than under continuous flooding. These findings might be due to the increasing mycorrhizal colonization in roots, which leads to the synthesis of more phospholipids for fungal membrane formation of hyphal branches in roots under intermittent flooding [46]. The AMF 0.2 cm mesh treatment under intermittent flooding decreased the neutral lipid concentration in roots compared with that of the NM treatment under intermittent flooding and the AMF 0.2 cm mesh treatment under continuous flooding (Figure 6b), suggesting that neutral lipids synthesized in mycorrhizal roots might be transported to extraradical mycelium [46,47].

### 4.3. Effects of Submergence and Arbuscular Mycorrhizal Fungi on Root Morphological Traits

AMF forms a symbiosis with vascular plants and is an ancestral, conserved trait [1,13]. Plant root morphological traits are strongly related to symbiosis with AMF [48]. Total lateral root length, S-type lateral root length, L-type lateral root length, branching root density, and root length density were increased following AMF 25 μm treatments, especially under intermittent flooding (Figure 5a,b,e,f), indicating that AMF addition stimulated the growth of rice roots to interact with AMF (Figure S1). There was no significant difference in mean root diameter between AMF 0.2 cm mesh treatment (with low mycorrhizal colonization) and NM treatment under continuous flooding. However, AMF 0.2 cm mesh treatment led to relatively high mycorrhizal colonization and increased mean root diameter compared with NM treatment under intermittent flooding (Figure 5d and Table S2). These findings suggest that the mean root diameter was strongly correlated with the extent of mycorrhizal colonization [48,49]. AMF 0.2 cm mesh treatments increased L-type lateral root length and root biomass (Figure 5b,g), which implies that longer-living and thicker roots were associated with more carbohydrate storage and growth [50]. An increase in lateral root length, surface area, volume, and root length density following AMF 0.2 cm mesh treatments (Figure 5 and Figure S1) is conducive to both deeper and shallower soil exploration [51]. Interestingly, continuous flooding applying to the AMF 0.2 mesh cm treatment, which led to low mycorrhizal colonization, developed a more derived ‘opportunistic strategy’, in which increased S-type lateral roots enabled plants to leverage photosynthetic carbon more efficiently for soil exploration [48].

### 4.4. Acclimation Strategy of Mycorrhizal Rice to Submergence

To acclimate to the flooding stress, rice has evolved a ‘submergence metabolic adaptation strategy’ by regulating sugar and energy production to program rice growth, and a ‘submergence escape strategy’ by promoting shoot elongation to allow rice shoots to rapidly rise above the floodwater surface [29]. *OsSUB1A* is a major quantitative trait locus conferring the submergence tolerance of rice by enhancing the action of gibberellic acid to promote shoot elongation [52]. In the NM treatments, a lower *OsSUB1A* expression in the rice stem, and a greater height of the rice plant under continuous flooding compared to those under intermittent flooding (Figure 3a and Figure 4b), imply continuous flooding-triggered rice stem elongation by mediating *OsSUB1A* expression to allow the shoot to rapidly rise above the floodwater surface to acclimate to the submergence [53]. Additionally, continuous flooding led to a lower starch concentration in NM treatments compared with intermittent flooding. Notably, the total soluble sugar concentration in roots under continuous flooding was approximately half that observed under intermittent flooding (Figure 7a,b). These observations indicate that rice plants under continuous flooding carried out a ‘submergence-escape strategy’ at the expense of carbohydrate metabolisms in roots for shoot growth, which led to low sugar and starch concentrations in roots [29]. Rice plants under continuous flooding with slower photosynthesis rates had a lower mycorrhizal colonization rate in roots than those under intermittent flooding (Figure 4a and Table S2), implying a possible reduction of carbon allocation from rice to AMF under continuous flooding [8]. Therefore, the limitation of carbon sources caused by flooding might limit the AMF colonization in rice roots.

**Figure 8:**
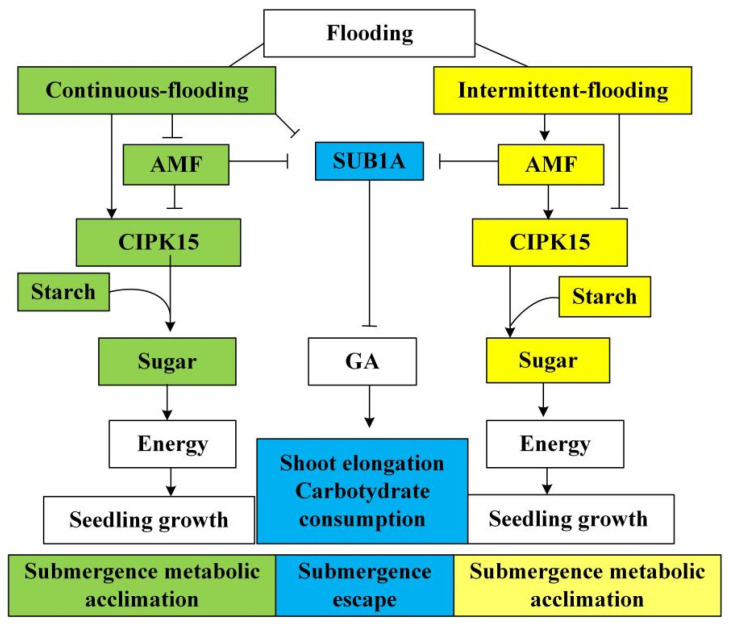
A model for *SUB1A-* and *CIPK15*-mediated acclimation to flooding stress for mycorrhizal rice under flooding conditions.

We propose a possible model for *OsSUB1A-* and *OsCIPK15*-mediated responses to submergence stress for mycorrhizal rice (Figure 8). Following the AMF 0.2 cm mesh treatments, *OsSUB1A* expression was down-regulated in stems, resulting in higher plant height compared with the NM treatments under different flooding conditions (Figure 3a and Figure 4b). These findings suggest that mycorrhizal rice acclimated to different intensities of submergence by triggering the ‘submergence-escape strategy’. The AMF 0.2 cm mesh treatments increased photosynthesis and led to an increase in total soluble sugar concentration in rice roots [8,54]. However, a ‘submergence metabolic-acclimation strategy’ was inhibited in AM 0.2 cm mesh treatment by suppressing starch consumption in rice roots through down-regulating *OsCIPK15* expression (Figure 3b and Figure 7b) compared with NM treatment under continuous flooding [29]. These findings demonstrate that AMF activated a ‘submergence-escape strategy’ but inhibited a ‘submergence metabolic-acclimation strategy’ of rice to acclimate to the submergence under continuous flooding. It also implies that mycorrhizal rice under continuous flooding redistributed soluble sugar and starch to enhance the growth of the aerial parts of rice that had access to oxygen, which was transported to roots to sustain the oxygen requirements of both rice roots and AMF [55]. However, the above strategy might decrease the energy metabolism of rice roots and affect ATP production because the synthesis of symbiotic substances, i.e., lipids, requires a large amount of ATP in rice roots, which might be one key reason for inhibiting the extent of the symbiosis between rice and mycorrhizal fungi under continuous flooding [43]. Also, intermittent flooding up-regulated *OsCIPK15* expression and enhanced starch consumption in rice roots following AMF 0.2cm mesh treatment (Figure 3b and Figure 7b). These findings indicate that the increasing mycorrhizal colonization in rice roots under intermittent flooding simultaneously activated a ‘submergence metabolic-acclimation strategy’ and a ‘submergence-escape strategy’, reducing competition for carbon between rice roots and AMF to acclimate to the submergence stress.

## 5. Conclusions

Increasing submergence intensity decreased phospholipid concentration in mycorrhizal roots, suppressed the transcription of the rice gene *OsD14L* involved in pre-symbiotic fungal perception, and reduced mycorrhizal colonization and the neutral lipid concentration in mycorrhizal roots. AMF inoculation enhanced LR growth, and the extent of mycorrhizal colonization was associated with the growth of LRs. Rice plants under continuous flooding with low mycorrhizal colonization exhibited an ‘opportunistic strategy’ by increasing S-type LR, which enables plants to more efficiently leverage photosynthetic carbon for soil exploration. Mycorrhizal rice mediated the expression of *OsCIPK15* and *OsSUB1A*, thereby activating a ‘submergence-escape strategy’ under continuous and intermittent flooding, and activating a ‘submergence metabolic-acclimation strategy’ under intermittent flooding, which was inhibited under continuous flooding to acclimate to the submergence.

## Data Availability

The datasets generated during and/or analyzed during the current study are available from the corresponding author upon reasonable request. The data are not publicly available due to the data used to support the findings of this study are currently under embargo while the research findings are commercialized.

## Supplementary Materials

The following supporting information can be downloaded at: https://www.mdpi.com/article/10.3390/plants13141908/s1, Table S1: primer sequences; Table S2: Concentration of dissolved oxygen (DO) and root colonization by AMF under different treatments; Figure S1: Effects of arbuscular mycorrhizal fungi (AMF) on root morphology of rice under different flooding conditions. Controls received no inoculum that AMF chamber with nylon sheet preventing plant roots from passing through and preventing diffusion of substances (NM); AMF chamber with nylon mesh with a diameter <25 μm preventing plant roots and mycorrhizal hyphae from passing through, but allowing diffusion of substances (AMF 25 μm); AMF chamber with nylon mesh with a diameter >0.2 cm allows plant roots and mycorrhizal hyphae to pass through and also substances to diffuse (AMF 0.2 cm). Continuous-flooding (Con); intermittent-flooding (Int).
