# Supplementary material for: Arbuscular Mycorrhizal Fungi Mediate the Acclimation of Rice to Submergence
Source: Plants (Basel). 2024 Jul 10;13(14):1908. doi: 10.3390/plants13141908 (PMC11280967; doi:10.3390/plants13141908)
Supplement: Supplementary file 1 [file plants-13-01908-s001.zip › plants-3066668-supplementary.pdf]

Supporting Information

Arbuscular Mycorrhizal Fungi Mediate the Acclimation of Rice to Submergence

Yanggui Xu [1,2,3], Yuting Tu [1,2,3], Jiayi Feng [4], Zhiping Peng [1,2,3], Yiping Peng [1,2,3] and Jichuan Huang [1,2,3,*]

[1] Institute of Agricultural Resources and Environment, Guangdong Academy of Agricultural Sciences, Guangzhou 510640, China; xuyanggui@gdaas.cn (Y.X.); tuyuting@gdaas.cn (Y.T.); pengzhiping@gdaas.cn (Z.P.); pengyiping@gdaas.cn (Y.P.)
[2] Key Laboratory of Plant Nutrition and Fertilizer in South Region, Ministry of Agriculture, Guangzhou 510640, China
[3] Guangdong Key Laboratory of Nutrient Cycling and Farmland Conservation, Jinying Road, Guangzhou 510640, China
[4] Guangdong Eco-Engineering Polytechnic, Guangzhou 510520, China; leave4s@126.com
* Correspondence: huangkuang_2002@aliyun.com

Number of pages:3

Number of tables:2

Number of figures:1

Figure legends

Fig. S1 Effects of arbuscular mycorrhizal fungi (AMF) on root morphology of rice under different flooding conditions. Controls received no inoculum that AMF chamber with nylon sheet preventing plant roots from passing through and preventing diffusion of substances (NM); AMF chamber with nylon mesh with a diameter ＜25 μm preventing plant roots and mycorrhizal hyphae from passing through, but allowing diffusion of substances (AMF 25 μm); AMF chamber with nylon mesh with a diameter ＞0.2 cm allows plant roots and mycorrhizal hyphae to pass through and also substances to diffuse (AMF 0.2 cm). Continuous-flooding (Con); intermittent-flooding (Int).

Table S1 primer sequences

Genes	primer sequences	
OsCERK1	5′-GCCTCCAGAGTATGCTCGAT -3′	5′-GAGGGCCTCCTCAAACAGAT-3′
OsD14L	5′-ACCACTTGTCACCGTACCAT -3′	5′-TGCTTGTGCAGGTACTCAGA-3′
OsSUB1A	5′-CACGTCTCCTCCGGTTATCA-3′	5′-TTTGCCGCTAGATCGGTTTG-3′
OsMYR	5′-TGGTTGCAAGGTTTGGGATG-3′	5′-GAGGAGCAAAGTAGCAACGG-3′
OsCIPK15	5′-GCGTGGAAAGCTTACAGAGG-3′	5′-CTGAAAGCGCACTCAATCCA-3′
OsUBI	5′-CAAGATGATCTGCCGCAAATGC-3′	3′TTTAACCAGTCCATGAACCCG-3′

Table S 2 Concentration of dissolved oxygen (DO) and root colonization by AMF under different treatments

Indices	Con			Int		
	NM	AMF 25 μm	AMF 0.2 cm	NM	AMF 25 μm	AMF 0.2 cm
DO ($mg \cdot L^{-1}$)	2.34±0.17b	-	2.07±0.43b	3.51±0.14a	-	2.60±0.09b
Colonization (%)			8.0±0.6			27.9±1.6*

* P < 0.05, t-test.

Fig. S1

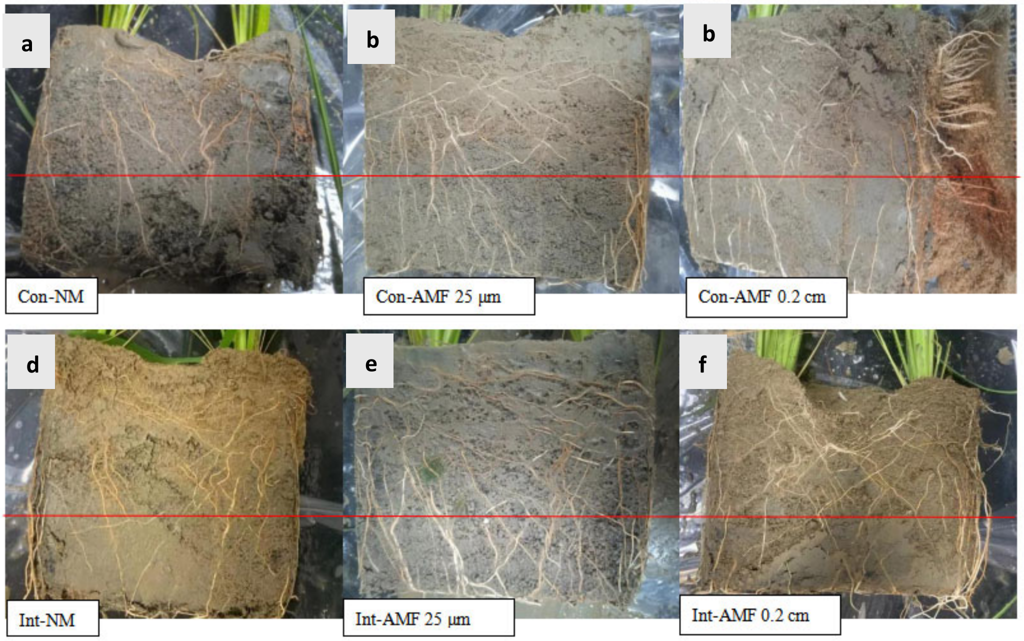

a
Con-NM
b
Con-AMF 25 μm
b
Con-AMF 0.2 cm
d
Int-NM
e
Int-AMF 25 μm
f
Int-AMF 0.2 cm
